# “What If It Was Your Dog?” Resource Shortages and Decision-Making in Veterinary Medicine—A Vignette Study with German Veterinary Students

**DOI:** 10.3390/vetsci10020161

**Published:** 2023-02-17

**Authors:** Kirsten Persson, Wiebke-Rebekka Gerdts, Sonja Hartnack, Peter Kunzmann

**Affiliations:** 1 Applied Ethics in Veterinary Medicine Group, Institute for Animal Hygiene, Animal Welfare and Farm Animal Behaviour, University of Veterinary Medicine Hannover, Foundation, Bischofsholer Damm 15, Geb. 116, 30173 Hannover, Germany; 2 Institute for Biomedical Ethics, University of Basel, Bernoullistrasse 28, 4056 Basel, Switzerland; 3 Section of Epidemiology, Vetsuisse Faculty, University of Zurich, Winterthurerstr. 270, 8057 Zurich, Switzerland

**Keywords:** veterinary ethics, moral reasoning, vignettes, teaching veterinary ethics

## Abstract

**Simple Summary:**

The here presented study was part of a survey on ethical decision making among veterinary students at the University of Veterinary Medicine Hannover, Foundation. The students were confronted with challenges in a situation in veterinary practice. Firstly, in the situation, two patients needed a medication but only one dosage was available in the veterinarian’s supply. The students based their decision regarding the first challenge, who should (not) get the medication, on the patients’ age, general health, life expectancy, the patient owners’ wellbeing, and their general knowledge on situations in veterinary practice. Secondly, the students were asked what would change if one of the patients was their own dog. They reacted in four different ways to the question. (1) For a professional, this should not make a difference; (2) they would most likely give the medication to their own dog; (3) they would give the medication to the other dog; and (4) they avoided a decision. Finally, the students judged a list of possible solutions to the dilemma on a scale (from very poor to very good). They preferred those solutions that focused on the animal’s wellbeing to those that focused on the owners’ wellbeing. Overall, it turned out that in situations of limited medication, students make their decisions for very different reasons, and that a guideline for veterinarians to make decisions in such situations is still missing.

**Abstract:**

The here presented vignette study was part of a survey on ethical judgement skills among advanced veterinary students at the University of Veterinary Medicine Hannover, Foundation. The vignette describes a fictitious dilemma in veterinary practice due to medication supply shortages. First, the students should make an ethically justified decision: who of the two patients in the waiting room gets the last dosage of a medication. Important factors were the animal patients’ characteristics (age, state of health, life expectancy), the patient owners’ wellbeing, and context-related criteria. Second, the students were asked for decisional changes if one of the patients was their own dog. They reacted in four different ways: (1) for a professional, this should not make a difference; (2) most likely being “egoistic” and preferring their own dog; (3) giving the medication to the other dog; and (4) avoiding a decision. Finally, the students judged a list of possible solutions to the dilemma on a 9-point scale. They preferred patient-related criteria to patient-owner-related criteria in this task. In the overall results, it became obvious that no “gold standard” or guidelines for situations of medication shortages exist, yet, which presents an important subject for future research and veterinary ethics teaching.

## 1. Introduction

It has been observed in recent years that the general public has become increasingly interested in the welfare of animals. This growing awareness not only concerns farm animals, but also companion animals such as dogs, cats, and horses [1]. To the same extent, it can be observed that the moral status of animals is changing and, especially in veterinary medicine, therapies are becoming possible and are also demanded and financed by animal owners, which were previously mostly reserved for human individuals [2]. Animals are no longer seen only as “pets”. In large parts of western societies they are often also ascribed the status of a “friend” or “family member” [3]. With new technologies and medications available, further aspects that accompany human medical practice may enter veterinary practice. One of them, the threat of resource shortages, will be dealt with in the here presented study with veterinary students.

Due to the knowledge and skills veterinarians have acquired during their training, they often see themselves as being in a special position of responsibility towards humans and animals. This responsibility is also attributed to veterinarians from external sources [4]. In practice, veterinarians are frequently caught between the individual interests of the owners and the presumed individual interests of the animals. Bernard E. Rollin identified the question of whether a veterinarian’s primary obligation is to the animal or to the animal owner as being the “fundamental problem” [5] in veterinary medicine already in 1988 and thus established the basis for the model of the Veterinary Triad [5,6].

Various studies have been conducted on stress levels among veterinarians and these showed that large parts of practicing veterinarians experience their profession as very stressful and morally demanding [7,8,9,10,11,12,13,14,15,16,17,18,19,20,21,22,23,24]. Primary stress factors mentioned were long working hours and on-call duties, but also fear of treatment errors, customer complaints, and pet owners’ claims that veterinarians are the experts in all areas of veterinary medicine. In addition, animal deaths were mentioned, whether from disease or euthanasia, social loneliness, lack of support in decision-making, and ethical challenges in general. A survey of small animal veterinarians in the USA identified ethical dilemmas as a major cause of work-related stress [7].

Accordingly, the conflicts veterinarians are confronted with in their everyday professional life can be of different nature—social or financial, but also moral [7,8,9,10,11,25,26,27,28,29]. Yeates (2009) was the first to analyze ethical conflicts in veterinary practice. He stated that especially with the increasing medical treatment options of veterinarians, the responsibility towards patients, animal owners, colleagues, and society also grows [4]. The fact that veterinarians do not always agree with the procedures of colleagues due to the abundance of treatment methods and that the concept of acting lege artis includes more and more methods, some of which differ greatly from one another, leads to an increasing need for ethical orientation. 

All of these study results document the need to prepare veterinary students for ethical challenges in practice. A recent study from North America shows that the cornerstone for stress and depression is already laid during university studies. Among the students surveyed, 49% reported having at least a moderate level of stress, and 66% showed symptoms of mild to moderate depression [17]. For this reason, veterinary medical education institutions were already called to account in 2013. In the context of the Federation of Veterinarians of Europe & European Association of Establishments for Veterinary Education’s (FVE & EAEVE) Report on Veterinary Education in Animal Welfare Science, Ethics and Law, the veterinary curriculum should offer a continuous examination of ethical questions that could be relevant to the later professional situations of veterinary students [30].

In response, the German Federal Ministry of Education and Research (BMBF) funded two successive projects on “Teaching veterinary, clinical and ethical skills” (FERTHIK I and II). [31] These projects were carried out consecutively at the University of Veterinary Medicine Hannover, Foundation, Hannover, Germany (TiHo). In particular, teaching in ethics was implemented. As part of this project, Germany’s only professorship for applied ethics in veterinary medicine was established in 2015 and corresponding teaching content was integrated into the curriculum [32]. The education in veterinary ethics at the TiHo is not only theory-based, but is also in the form of the concept of “critical guidance” [33]. A special focus is always on the practical applicability of ethical tools and methods. Especially the methodology of applied ethics with a bottom-up and top-down approach, i.e., the development of principles from individual cases and the application of these principles to further individual cases, represents a cornerstone of teaching veterinary ethics [34,35]. 

Finally, within the framework of the FERTHIK II project, a review of the moral judgement skills of veterinary students should take place. Although veterinary ethics is required in the veterinary curriculum in Germany and part of the First Day Competencies expected of a trained veterinarian according to the FVE and EAEVE, knowledge about tools to assess moral judgement skills is sparse [30]. To put it in Vettical’s [36] words: “Further investigation and dissertation on veterinary practice related ethical issues may perk up veterinary instruction and paraphrase of ethics assumption and way of thinking into functional practice.“ (p. 746). The present article aims to contribute to bridging this gap.

The here presented vignette study was part of a survey on ethical judgements among veterinary students at the TiHo in their fourth year. They had the opportunity to attend all ethics lectures, seminars, and block courses that were established in the new curriculum (for more details on the ethics curriculum see [32]).

The vignette describes a fictitious scenario in veterinary practice and introduces two aspects that call for an ethically justified decision. Two dogs suffering from intestinal cancer and their owners are sitting in the waiting room. The veterinarian has a new medication for intestinal cancer in dogs. However, only one dosage is available due to supply shortages. The first and obvious question is: who should be given the medication? Some additional information on the dogs (age and general health status) and their owners (living circumstances, time available for the dog, further companion animals) is given that might influence the decision. The second question tries to involve the participants personally in the scenario: what if the older dog patient was their own dog? While it is not uncommon that patient owners ask their veterinarian, “What if it was your pet?” this usually comes up in situations of different therapy options for the one animal when the patient owner is looking for help in the decision-making process [37]. In our case, the veterinary students should rather elaborate on the factors that influence their decision, including unexpected personal involvement.

Resource scarcity in the sense of a shortage of medicines and vaccines is not a common phenomenon in Germany. Nevertheless, it regularly happens that vaccines or medicines are not available to the usual extent and are also not available at short notice in other European countries or even worldwide [38,39,40,41]. Especially during the corona pandemic and afterwards, pet owners and veterinarians experienced supply problems with medicines and vaccines. A current example is the availability of vaccines against the Equine Herpes Virus (EHV). Due to the herpes outbreak among sport horses in Valencia (Spain) in 2021, vaccination against EHV was introduced as a core vaccination for sport horses in Germany as of 1 January 2023 [39]. In the preliminary discussions, it was argued that the vaccines against EHV were not available at all times in the past and that only strictly limited doses of the vaccine would have been available to veterinarians [40]. 

Ethics teaching should prepare veterinary students to use and name decision making criteria and, ideally, to develop a professional attitude that leads to consistent ethical judgements. The aim of the vignette study was, firstly, to investigate the participants’ skills to identify stakeholders and the ethical conflict, as well as the patterns of their decision-making and justifications. For a more detailed discussion of the classification of different attitudes among our participants, see [32]. Secondly, the focus of this particular vignette was on their reactions to an unconventional problem and to the challenge of personal involvement. Given the recent occurrence of supply shortages in human and veterinary medicine, decision making criteria for such cases should be investigated and sharpened, even though ethics teaching might not have a focus on this specific issue, yet.

## 2. Materials and Methods

For a detailed description of the complete study, please see [32]. The survey was pilot tested among members of the institute (veterinarians, agricultural scientists, and philosophers) and modified according to their feedback. The animal welfare officer of the TiHo confirmed that the TiHo’s ethical requirements for student participation in studies in the form of surveys were met in March 2020.

In summer 2020, the survey was made available online (LimeSurvey GmbH, Hamburg, Germany, www.limesurvey.org (accessed on 2 January 2023)) to a 262-student cohort in their fourth year of veterinary education. The constraints due to the pandemic made it impossible to gather the students for this purpose, as originally planned, in an exam-like situation and hand out a printed version of the survey. Rather, online participation was facultative, advertised via email. After several email reminders and a data collection time of approx. 16 weeks, the return rate was ca. 22% (*n* = 87).

In the survey, four fictional scenarios were presented, one of which is discussed in this article. The first three scenarios dealt with common challenges in veterinary practice (compliance issues in a farm animal case, a conflict due to an expensive treatment in small animal practice, and an animal welfare issue in a jumping horse scenario). The analysis and discussion of the corresponding results are published elsewhere [28]. The here presented fourth scenario asked the students to think about a rather unusual resource allocation problem, combined with a question regarding their general professional attitude. 

The scenario reads as follows (translated from German): 

“There are delivery shortages for a new medication against intestinal cancer in dogs. The next delivery is not due for several weeks. You only have the amount necessary to treat one dog—but there are two patients in the waiting room that would need the medicine urgently. 

Patient 1 is a two-year-old poodle and she was never seriously ill until the diagnosis. She lives in a family with two children and two adults and is alone half the day. Without the administration of the drug, her condition would deteriorate rapidly and she might not survive the wait for the next delivery.

Patient 2 is a nine-year-old terrier who lives with a younger dog with a pensioner. The man is a little frail but devotedly cares for his dogs around the clock. Without the administration of the drug, the dog’s condition would rapidly deteriorate and he might not survive the wait for the next delivery”.

We asked the participants:

1. What are the ethical conflicts in this case?
2. Who is involved in the conflict (stakeholders)?
3. What information is important? Do you need more information?
4. Which dog would you give the medication to? And why?
5. How would the situation change if patient 2 was your own dog?

After filling in the free-text answers, we presented a list of six statements and asked the participants to indicate their agreement with the statements on a scale from one (very poor) to nine (very good). To avoid any influence of the list of statements on the free-text answers, there was no “back” button in the survey. The statements suggested potential solutions to the case and read as follows:

- You toss a coin and administer the medicine to the dog that “wins”.
- You choose the poodle because statistically she will still have a longer lifespan than the older terrier.
- You choose the terrier because he receives better and continuous care and therefore has a better chance of recovery.
- You choose the poodle because an entire family is affected and not just an elderly person who has another dog.
- You openly explain the problem to both owners together and ask them to discuss the decision on their own.
- You have a joint discussion with the owners until a decision is reached that you all agree with.

Answers were exported in Microsoft^®^ Excel (Version 2016). One member of the team (KP) conducted the descriptive statistical analysis. Core quotes, i.e., statements that either represented an opinion that was frequently mentioned or an exceptional, uncommon point of view, were collected, translated verbatim, and are presented in the analysis. The figures were produced with Excel and R.

## 3. Results

Firstly, we present the results of each free-text answer and secondly, the results of the scale task.

### 3.1. What Is the Ethical Conflict?

Several participants explicitly reported this to be a challenging decision. First, a weighing process was necessary. Many students identified criteria for each patient: the poodle is younger, the terrier older. The poodle lives with a family who is absent half of the day. The terrier can be taken care of by his owner full-time. The members of the family might be more alert and mobile and better equipped to take care of a sick dog. The elderly patient owner might, however, be willing and be able to sacrifice more time and effort for his dog, as he might not have as much other company and distractions as the family. The difference the loss of each dog makes is equally hard to compare. In the one case, several family members would grieve. In the other case, it is mainly the one owner. However, the loss might be more profound to him as he does not have family members around and his life might be more focused on the dog. One student ascribed more “emotional value” of the dog to the elderly man. Additionally, there is the other dog living with the elderly man, who would suffer from the loss, too, but at the same time comfort his owner in his mourning. 

Several students described the problem as having to ascribe more value to one dog’s life than to the other dog’s, as having to judge which dog “earns” the treatment more, or, more generally, as the burden of having to decide about life and death. Many participants answered with a question to this question, e.g., “Who may live?”, “Who should be saved?”, “Who is more worthy of a therapy?”, “How can I decide here?”. At the same time, one student concluded that “it is not my job as a veterinarian to decide on the question which animal has a greater value”. A few answers emphasized that this was not only a decision concerning the patient’s life, but also that of the patient’s owner, which points toward the much-discussed Veterinary Triad ethics between patient, patient owner, and veterinarian [5,6,42,43,44]. 

### 3.2. Who Is Involved in the Conflict (Stakeholders)?

**Figure 1:**
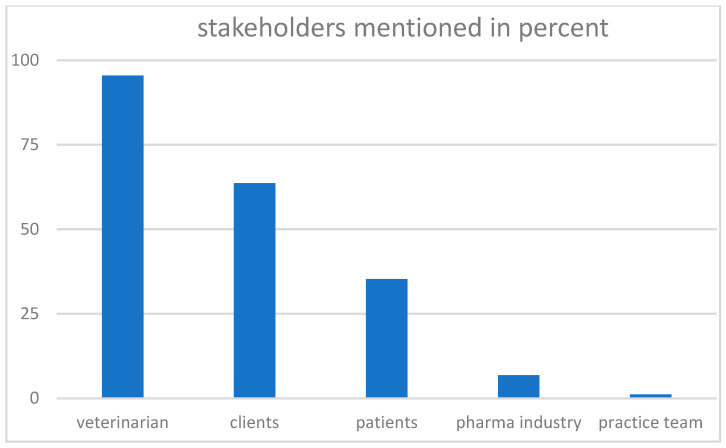
Answers to the second question, “Who is involved in the conflict (stakeholders)?” in percent.

For an overview of the answers to this question, see Figure 1. In line with the above-reported dominant answer that this conflict was mainly an inner decision-making challenge for the veterinarian, our participants identified the latter as the main stakeholder. 

The students were familiar with the prominent Veterinary Triad of animal—patient owner—veterinarian [2,3,4] and mentioned all three stakeholders. However, the patient owner was named as a stakeholder by more than 60% of the students, whereas the animal was only mentioned by a third of all participants. Those who looked beyond the narrow setting in the vignette (7%) also pointed out that the pharmaceutical industry was involved in the conflict.

### 3.3. What Information Is Important? Do You Need More Information?

Most students referred to the information that was already given in the vignette as necessary for their decision. They often mentioned the prognosis, which was roughly summarized in the scenario: “Without the medication, her/his condition would deteriorate rapidly and she might not survive the wait for the next delivery”. However, the chances for complete healing were not given, nor were potential side effects, which the students also asked for. Additionally, they wanted to know more about the general health, stage of the cancer, and potential comorbidities of the two dogs, in particular of the nine-year-old dog who can be considered elderly. This also included characteristics of the dogs like their “will to live”.

Besides, participants pointed out several ways to try to get the medication or alternative medication:

- Asking colleagues to help out or to take over the case,
- Looking for the same active agent in human medicine and ordering a medication for humans instead,
- Offering older/alternative and potentially less effective medication (given that in the vignette we wrote about a “new medication”),
- Searching for other ways of treatment like surgery or radiation therapy for one of the dogs,
- Trying to split the medication between the two dogs so every dog could receive a lower dosage,
- Thinking about palliative treatment.

Several students asked which dog had had the earlier appointment, had been higher on the waiting list or had entered the practice before the other, suggesting that, all other things being equal, this might be their final criterion when making a choice.

Furthermore, our participants needed more information on the animal owners that was not given in the vignette. For example,

- Who can afford the treatment?
- Can the family find a solution to take care of the dog while they are not at home?
- Does the elderly man have a support system he can activate?
- What is the animal owners’ opinion regarding the problem?
- What is the animal owners’ general attitude towards life?
- Who could better deal with the loss?/How close is the human-animal bond in both cases?

### 3.4. Which Dog Would You Give the Medication to? And Why?

**Figure 2:**
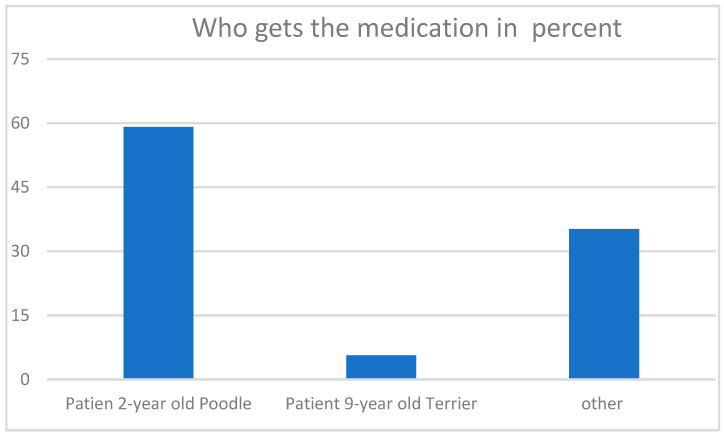
Answers to the third question “Which dog would you give the medication to? And why?” in percent. The answer “other” is further specified in the text below.

As presented in Figure 2, almost 60% of the participants decided to give the medication to patient 1, the poodle. Circa 5% opted for the terrier, patient 2, and about a third suggested other solutions or refused to decide.

The students explained that their reasons for choosing the poodle had mainly to do with her higher life expectancy. Some additionally deduced a better general state of health and a lower risk for comorbidities compared to the older dog, or, even a better prognosis. A couple of participants argued that the older dog would be better taken care of which is why the younger animal was in greater need of the medication. In addition to these patient-related reasons, students justified their choices with a focus on the patient owners. A few respondents mentioned that more patient owners would grieve if the poodle died. One student argued that the elderly patient owner’s second dog might “give him comfort in the time of trouble” if the sick dog died. Furthermore, a participant wondered if the elderly man might be overstrained with having to care for one sick animal and another dog. A different argument applied to the triage rules in times of COVID-19 when younger patients should be preferred in case of medication shortages. 

The few students who chose to give the medication to the terrier referred to patient owner-related arguments as opposed to the patient’s characteristics. They stated that the elderly man might be less flexible to look for the medication in another veterinarian’s practice or even abroad. Moreover, the students argued that the patient owner’s “own mental and psychological condition depends on the life of the dog” or that the dog was a more central friend for the elderly man than for the family. Similarly, one student assumed that “the family has enough distractions (work, school) and they have had the dog for two years or less, so the bond may not be as intense yet”. Comparing the characteristics and well-being of the two dogs, some students concluded that it might be easier to bridge the time until the medication was available again with an alternative for the younger dog due to the potentially worse overall state of health of the older animal. In contrast to that, a few respondents pointed out that a young poodle getting cancer suggested an underlying condition or a worse general state of health in the two-year old dog, which is why the terrier should be preferred. One student even postulated that the terrier had a “more meaningful life” in their opinion. 

Reasons for avoiding the decision were manifold. Several students explained that they would have sent one patient to a different practice/clinic and called the situation unrealistic. Most likely referring to their own experience in veterinary practice, they stated that the medication should be available somewhere else. To circumvent the problem, some participants suggested that dosage could be reduced, i.e., the medication could be split between the two patients. 

Several participants would give the medication to the patient with the earlier appointment (one student referred to the criteria for organ donation). It was suggested a few times to discuss with the patient owners and see whether someone was prepared to voluntarily do without the medication. A few participants declared that they were not able to decide due to a lack of information. One participant emphasized that the shortage was not their fault so it was not up to them to find a “fair” solution in this impossible situation. A few times, the ability to afford the treatment was mentioned as an additional important point for clarification. Chance/tossing a coin was suggested by a few students.

### 3.5. How Would the Situation Change If Patient 2 (the Terrier) Was Your Own Dog?

The students’ reactions to this question can be categorized in four groups. 

The first emphasized the objective and rational argument that it must not make a difference if the dog was their own and any deviation due to a consideration of personal needs and feelings would be unprofessional. One student explicitly wrote, “I would try and make a rational decision as a VETERINARIAN (not as an owner)”. A few participants stated that there was some “correct” decision in their view resulting from the balancing of arguments and this should be their conclusion in any case. A few tried to differentiate between what they knew they should do (give the medication to the dog with the better prognosis) and “if in reality I would actually sacrifice my own dog. I dare doubting that to be honest.”

The second group of participants was inclined to admit that they would decide “egoistically”, “subjectively”, or “emotionally”, and therefore give the medication to their own dog. They occasionally expressed awareness of this being a wrong or unprofessional decision, but, as one participant put it, “a veterinarian is human, too”, or, in the words of another student “if it is my own dog, unfortunately, rationality no longer comes into play”. One participant suggested that distance was an important factor in other moral decisions, too, and that it was, for example, considered morally acceptable to prefer relatives to strangers in forced decisions.

A third group, and that included several participants who had opted for giving the medication to the terrier in the previous question, would under these circumstances change their decision and give the medication to the younger dog (the poodle). They argued that, being vets, they would be able to take care of the suffering animal in a much better way than the family (or the elderly man) and could make him as comfortable as possible until the new medication arrived. In addition, some stated that they could cope better with the loss than the elderly man (in the original scenario) or the family (in direct comparison). Two participants brought up the unlikely idea that “due to my miscalculation of my medicine cabinet, my bad conscience would tempt me to treat the poodle”.

A fourth group, again, tried to avoid the decision-making. Several participants suggested referring the other dog to a colleague. Some argued that they would not be in a position like that at all because they would have taken care of the dog and would have obtained the medication much earlier. Many students explained that they would nonetheless be unable to decide in this case due to a lack of information (on prognosis, the type of disease, comorbidity, etc.) and that would not change if it was their dog. Therefore, they would make the decision depending on further medical information like the first group. However, some admitted that in case of a “tie” they would opt for their own dog.

### 3.6. Judgement of Suggested Actions

**Figure 3:**
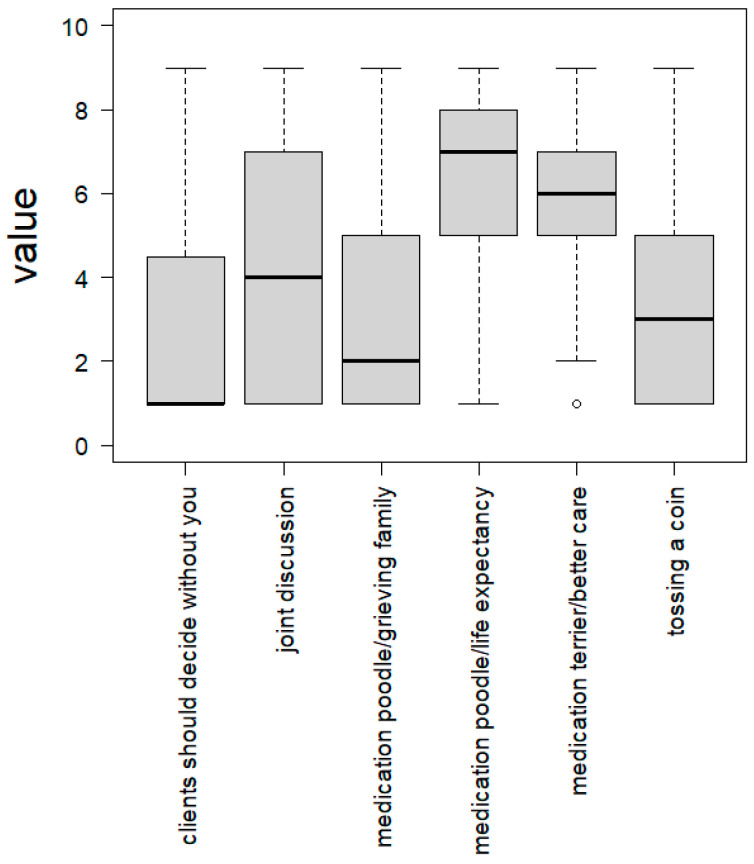
Answers for the six statements presenting different options for decisions, including medians. Judgement on a scale from 1 (very poor) to 9 (very good).

The students judged the provided options to solve the conflict as follows on a scale from 1 (very poor) to 9 (very good) (see Figure 3): They agreed the most (median 7) on giving the medication to the poodle because of her higher life expectancy. The second best option (median 6) presented the option to give the medication to the terrier, as he would be better taken care of. A joint discussion involving the veterinarian and the two patient owners was judged to be somehow acceptable (median 4), whereas tossing a coin received a lower agreement (median 3). The students did rather not agree (median 2) to justify giving the medication to the poodle with the grieving family in contrast to the elderly man who would grieve for the terrier, and they depreciated the option to let the patient owners solve the issue in a discussion without the veterinarian (median 1).

## 4. Discussion

The combination of being forced to decide, not having differentiating criteria to justify the decision, and the threatening deadly outcome for one of the patients made the decision explicitly challenging for the respondents. The free-text answers show that students found different strategies to cope with this problem.

- *Strategy 1: Patient-centered decision making*

Although quite a few students pointed out that they would need more details on the prognosis, the general health status of the patients, the progression of the disease, or the specifics of the medication, many justified their opting for the younger patient with the higher life expectancy and corresponding assumed better overall health status. Those opting for the older patient often argued similarly but came to a different conclusion: the younger dog was more likely to survive until the medication was available again, which was why the older one needed the dosage that was immediately available. 

Focusing on the patients’ interests, they might have internalized what Weich and Grimm [45] outlined as a role model: “clinical practice is guided by a normative dimension of the concept “animal patient” as an ethical ideal. In determining whether a veterinarian acts ethically, the norm of aiming at the health-related interests of the animal patient is decisive. Acting according to this norm constitutes good veterinary clinical care.” (p. 262). This attitude or role is known as the “animals’ advocate” [46].

While it should not be doubted that it is in the animal patient’s interest to be cured of intestinal cancer, opting for the younger dog for the reason of her higher life expectancy points towards a more complicated assumption. When one participant referred to triage rules in human medicine and suggested preferring younger patients, they implied that animals, like humans, might expect a certain life expectancy or might prefer to live as long as possible (for a more exhaustive discussion of age in human and veterinary medical decision making, see [47]). If a lower age correlates with a better general state of health, this presents, however, a medically relevant difference that serves as an argument to prefer the younger dog. On comparing the free-text answers to the statements with suggested solutions, it is striking that the respondents to a greater extent agreed to the two patient-centered solutions (medians were 6 and 7 of 9; see Figure 3). This result again underlines the dominant role model of the veterinarian as the animal’s advocate.

- *Strategy 2: Patient owner-centered decision-making*

Several arguments referred to the differences the veterinarian’s decision would make for the patient owners rather than for the patients. By no means does this imply that the students providing these arguments consider themselves less as animals’ advocates than those using strategy 1. It can be the case that the differences between the patients, especially regarding their interests and “claims” to obtain the medication, are perceived as insufficient for a decision. Turning to the patient owners is a comprehensible next step. Other than the prominent advocacy for the animal, weighing the interests of different patient owners is a rather uncommon or at least not an outspoken decision-making process in veterinary medicine. After all, the idea of a veterinarian telling the children of the poodle’s family that the terrier’s elderly owner would feel so lonely without him seems unusual. Given that the here presented case of resource allocation is also very rare in veterinary medicine, though, the patient owner-centered arguments cannot be excluded as less relevant per se. After all, the students’ awareness of and sensitivity for the patient owners’ needs and welfare are important goals of veterinary ethics teaching [30]. Similarly for the patient-centered strategy, several outcomes are possible. The fact that several people—some of them children—would grieve for the poodle compared to only one owner (and a partner dog, who is, however, rarely mentioned in this respect) for the terrier, is up against the fact that the family can be expected to have a fulfilled life full of activities even without their dog, whereas the elderly man might, for example, lose his daily routines and activities without a dog because he might be much more focused on the animal.

- *Strategy 3: Established ways of solving conflicts in veterinary practice*

About one third of the respondents were not willing to fully get involved in the scenario as a thought experiment. Many pointed towards usual procedures in veterinary practice that would circumvent the conflict we presented. Some highlighted the avoidable problem of scarcity. They suggested sending the patient to a colleague, calling several colleagues to still get the medication, or, if need be, to split the dosage so that both patients could be supplied for a short time. Additionally, the vignette framing the medication as “new” was understood in a way that an older medication could be available in the meantime. The alternative interpretation that the medication was an innovative approach in treating this type of cancer that way (i.e., without chemo- or radiation therapy) was not considered in those cases. The list of statements provided some suggestions that are not (or should not be) established in veterinary practice when it comes to involving patient owners. The idea of having a joint discussion with both patient owner groups was judged to be a comparatively good solution (4 of 9; see Figure 3). Buck passing, on the other hand, was not appreciated. Despite admitting that the decision was tough in this case, veterinary students obviously did not want to leave it to the patient owners to decide without themselves being involved.

- *Strategy 4: Arbitrary decision making*

Another way of reacting to the question can be attributed to an account of distributive justice. If the morally relevant properties of the two potential receivers of the scarce resource are equal, it might seem wrong to prefer one to the other. Following this argument, several participants suggested giving the medication to the dog who entered the practice first or was higher on the waiting list (a criterion practiced when allocating donated organs). Others considered tossing a coin, which was also a suggestion in the provided list of statements (see Section 3.6), leaving the decision to chance. All of these decisions provided answers to the questions that other students raised to express their inability to come to a decision in the given situation (see Section 3.1). 

The final question that would change if patient 2 was their own dog was meant to put the students in a position of merging professional and private arguments. This merging effect was reflected in the students’ answers. Some reacted as (private) companion animal owners, stating that their own dog would obviously be preferred. That way, they fell back on a patient owner-centered argument. Others clearly judged the situation professionally, stating that it should not make a difference whom a patient belonged to and they would administer the medication according to objective criteria. A third way of reaction presented an attitude of a veterinarian as a special kind of dog owner. With their additional knowledge and skills, those participants felt a special responsibility in the given situation, taking the burden of caring for an animal without the proper medication on their shoulders because they could not impose it on a “non-professional” dog owner. This argument pattern presents a vivid illustration of a “unit of care” approach, considering both the interests of patients and patient owners, and the team effort they can contribute to the process together. 

The overall reactions of the participants to this scenario suggest that resource shortages, a potentially manifest problem in European human and veterinary practice, have not been introduced thoroughly in veterinary ethics teaching. In human medicine, particularly in oncology, codes of conduct were developed by professional associations during the COVID-19 pandemic that can serve as guidelines when resources are scarce, especially for life-saving drugs. In this context, criteria were developed to enable physicians to make well-founded decisions in ethically extremely difficult situations. The American Society of Clinical Oncology recommendations [48], for example, implicitly mention factors that we similarly observed in veterinary students: “Ethical principles at the forefront of pandemic planning differ from patient-centered approaches that may be more familiar to oncologists.“ (p. 7). The students we interviewed based their assessment of the situation and their decision-making not only exclusively on the patient and his or her individual circumstances and criteria, such as age and chances of recovery, but also on the social environment in which the animal lived. Similarly, the guidelines address, for example, preference for one’s animal in the context of “fairness”: “Resources should be allocated based on ethically-relevant differences among individuals, free from unjustified favoritism and discrimination.” (p. 10). It is possible that such guidelines and codes of conduct from human medicine could be adapted and modified for veterinary medicine, although such extreme cases of resource scarcity are fortunately very rare.

Given that the data collection took place in 2020, the consequences of the pandemic for the pharmaceutical market were not yet as explicit when the students filled out the survey. Not having a gold standard for this case, a clear guideline they could refer to when being confronted with a dilemma situation presented a discomforting challenge which individual students solved in different ways. Further research in this thematic field is needed to develop decision-making support for veterinarians in similar real-life situations.

## Data Availability

The data presented in this study are available on request from the corresponding author. The data are not publicly available in order to keep the anonymity of our (small number of) participants.
